# Chronic Lymphocytic Leukaemia (CLL)-Associated Proliferative Glomerulonephritis With Monoclonal Immunoglobulin Deposits (PGNMID) Diagnosed in the Absence of Detectable Paraprotein

**DOI:** 10.7759/cureus.112008

**Published:** 2026-07-03

**Authors:** Filipa Rodrigues, Mariana Pais, Rafael Terencio, Rita Manso, Joana Silva

**Affiliations:** 1 Nephrology, Unidade Local de Saúde de Almada-Seixal, Almada, PRT; 2 Pathology, Unidade Local de Saúde de Amadora/Sintra, Amadora, PRT

**Keywords:** chronic lymphocytic leukemia (cll), kidney biopsy, monoclonal gammopathy of renal significance (mgrs), onco-nephrology, proliferative glomerulonephritis with monoclonal igg deposits (pgnmid)

## Abstract

Renal involvement in chronic lymphocytic leukaemia (CLL) is uncommon but clinically relevant. We report a man in his early 70s with known CLL who presented with non-nephrotic proteinuria, progressive kidney injury, and hyperkalaemia. Kidney biopsy revealed diffuse membranoproliferative glomerulonephritis with IgG kappa monoclonal deposits on immunofluorescence and electron microscopy, establishing a diagnosis of CLL-associated proliferative glomerulonephritis with monoclonal immunoglobulin deposits (PGNMID). Treatment with rituximab and chlorambucil resulted in significant improvement of both kidney function and proteinuria. This case highlights the pivotal role of kidney biopsy in CLL-associated nephropathy: it established a precise diagnosis in the absence of detectable circulating paraprotein, triggered clone-directed therapy, and demonstrated that meaningful renal recovery is achievable even in the context of acute kidney injury.

## Introduction

Chronic lymphocytic leukaemia (CLL) is the most prevalent adult leukaemia in Western countries, characterised by the progressive accumulation of functionally incompetent monoclonal B-lymphocytes in peripheral blood, bone marrow, and lymphoid tissues [1]. Its clinical course is highly heterogeneous: many patients remain asymptomatic and require no treatment for decades, whereas others develop progressive disease necessitating early systemic therapy [2].

Renal involvement is an underrecognised but clinically significant complication of CLL, arising through three distinct mechanisms: direct leukaemic infiltration of the renal parenchyma, immune-mediated glomerulonephritis, or therapy-related nephrotoxicity [1]. Although autopsy series suggest leukaemic infiltration occurs in up to 90% of patients at late stages, symptomatic kidney involvement is uncommon [3]. Among the immune-mediated mechanisms, the CLL clone may secrete a nephrotoxic monoclonal immunoglobulin that deposits within the glomeruli. When such a clone causes renal end-organ damage without meeting the haematological criteria for treatment, the resulting disorder is termed monoclonal gammopathy of renal significance (MGRS), a framework that reclassifies small, otherwise clinically indolent B-cell clones as pathogenic when they injure the kidney [4,5].

Proliferative glomerulonephritis with monoclonal immunoglobulin deposits (PGNMID) is one such MGRS-defining lesion. It is characterised by a proliferative, frequently membranoproliferative, glomerular pattern, with granular deposits of a single immunoglobulin isotype and light-chain restriction on immunofluorescence, and amorphous, non-organised electron-dense deposits on electron microscopy [1,5]. A diagnostic challenge particular to PGNMID is that the causative clone is often small and low-secreting; consequently, a circulating monoclonal protein is undetectable by serum and urine studies in a substantial proportion of cases, and the diagnosis rests on kidney biopsy with full immunofluorescence and electron microscopy characterisation [1,5].

This case is instructive for the onco-nephrology community for three reasons. First, it demonstrates that clinically significant monoclonal immunoglobulin-related kidney disease may be present in CLL even when serum and urine paraprotein studies are entirely negative. Second, it shows that the biopsy finding can directly change management: here, documenting symptomatic organ involvement fulfilled an International Workshop on Chronic Lymphocytic Leukaemia (iwCLL) criterion for active disease and provided the indication to initiate clone-directed therapy in a patient previously managed expectantly [2]. Third, it illustrates that meaningful renal recovery is achievable with treatment, even in the setting of established acute kidney injury. A low threshold for kidney biopsy incorporating immunofluorescence and electron microscopy therefore remains indispensable in CLL patients who develop unexplained renal dysfunction, such as a decline in eGFR, proteinuria, or an active urinary sediment [5].

## Case presentation

A 71-year-old man was referred to our Nephrology consult for evaluation of progressive kidney function decline, with serum creatinine rising from a baseline of 1.7 mg/dL to 3.3 mg/dL over approximately six months. He had a four-year history of CLL, characterised by 50% monoclonal B-cells with CLL phenotype, mutated immunoglobulin heavy chain variable (IGHV) region status, fluorescence in situ hybridization (FISH) deletion 13q, and normal karyotype (46 XY), that had not previously met iwCLL criteria for systemic treatment. Medical history included longstanding hypertension, benign prostatic hyperplasia, and absolute eosinophilia (1,210/µL), the latter considered a paraneoplastic manifestation; molecular testing for PDGFRB, FGFR1, and JAK2 rearrangements was negative, excluding a primary myeloproliferative aetiology. He was a former smoker with light alcohol consumption and no relevant occupational exposures. Current medications included tamsulosin, allopurinol 100 mg, and mirtazapine 15 mg. He was not receiving antihypertensive therapy at referral. On examination, blood pressure was 145/70 mmHg and heart rate 70 bpm. Bilateral pitting oedema of the lower limbs was present, with no palpable lymphadenopathy or organomegaly.

Laboratory investigations are summarised in Table 1 and the patient's clinical course, from baseline renal function through presentation, biopsy, treatment, and follow-up, is summarised chronologically in Table 2. Haematology revealed normochromic normocytic anaemia (haemoglobin, 10.5 g/dL) without nutritional deficiency; ferritin was 160 ng/mL with transferrin saturation 34%, and folate and vitamin B12 were normal. White cell count was markedly elevated at 62.1 × 10⁹/L with 87% lymphocytes, consistent with active CLL disease burden. Lactate dehydrogenase (LDH) was within normal limits at 211 U/L, arguing against tumour lysis or haemolysis. Serum creatinine was 3.3 mg/dL, corresponding to an eGFR of 22 mL/min/1.73m² (Chronic Kidney Disease Epidemiology Collaboration (CKD-EPI) 2021). Hyperkalaemia (potassium, 6.2 mmol/L) and hyperuricaemia (uric acid, 11.3 mg/dL) were present. Serum albumin was 4.0 g/dL and lipid profile unremarkable. Glycated haemoglobin (HbA1c) was 4.8%, effectively excluding diabetes. IgG was markedly reduced at 347 mg/dL, consistent with CLL-associated hypogammaglobulinaemia. Autoimmune workup was negative, including antinuclear antibodies (ANA), antineutrophil cytoplasmic antibodies (ANCA, both anti-myeloperoxidase (MPO) and anti-proteinase 3 (PR3)), complement levels (C3 and C4 within normal range), and cryoglobulins.

**Table 1:** Laboratory findings at presentation ALP, alkaline phosphatase; ALT, alanine aminotransferase; AST, aspartate aminotransferase; CKD-EPI, Chronic Kidney Disease Epidemiology Collaboration; CLL: chronic lymphocytic leukaemia; eGFR, estimated glomerular filtration rate; GGT, gamma-glutamyl transferase; HbA1c, glycated haemoglobin; HDL, high-density lipoprotein; IgG, immunoglobulin G; LDH, lactate dehydrogenase; LDL, low-density lipoprotein; PSA, prostate-specific antigen; PTH, parathyroid hormone; RPR, rapid plasma reagin; SLL: small lymphocytic lymphoma; 25-OH: 25-hydroxyvitamin D; MPO-ANCA: myeloperoxidase anti-neutrophil cytoplasmic antibody; PR3-ANCA: proteinase 3 anti-neutrophil cytoplasmic antibody; HBsAg: hepatitis B surface antigen; SLL: small lymphocytic lymphoma; CD: cluster of differentiation.

Parameter	Value	Reference range
Haematology and biochemistry		
Haemoglobin	10.5 g/dL	13.5-17.5 g/dL
Platelets	469,000/µL	150,000-400,000/µL
White cell count	62,100/µL	4,000-11,000/µL
Neutrophils	9%	40%-70%
Lymphocytes	87%	20%-45%
Ferritin	160 ng/mL	12-300 ng/mL
Transferrin saturation	34%	20%-50%
Folate	6.9 ng/mL	>3.0 ng/mL
Vitamin B12	279 pg/mL	200-900 pg/mL
Glucose	94 mg/dL	70-99 mg/dL
HbA1c	4.8%	<5.7%
Urea	109 mg/dL	10-50 mg/dL
Creatinine	3.3 mg/dL	0.7-1.2 mg/dL
eGFR (CKD-EPI 2021)	22 mL/min/1.73 m²	>60 mL/min/1.73 m²
Sodium	137 mmol/L	136-145 mmol/L
Potassium	6.2 mmol/L	3.5-5.0 mmol/L
Phosphorus	4.8 mg/dL	2.5-4.5 mg/dL
Calcium	8.2 mg/dL	8.5-10.5 mg/dL
Magnesium	2.9 mg/dL	1.7-2.4 mg/dL
Uric acid	11.3 mg/dL	3.5-7.2 mg/dL
AST	15 U/L	<40 U/L
ALT	10 U/L	<40 U/L
GGT	19 U/L	<55 U/L
ALP	85 U/L	40-130 U/L
Total bilirubin	0.3 mg/dL	<1.2 mg/dL
Albumin	4.0 g/dL	3.5-5.0 g/dL
LDH	211 U/L	120-246 U/L
Total cholesterol	157 mg/dL	<200 mg/dL
HDL	66 mg/dL	>40 mg/dL
LDL	82 mg/dL	<130 mg/dL
Triglycerides	52 mg/dL	<150 mg/dL
C-reactive protein	0.24 mg/dL	<0.5 mg/dL
PSA	2.69 ng/mL	<4.0 ng/mL
PTH	119 pg/mL	15-65 pg/mL
Vitamin D (25-OH)	8.3 ng/mL	30-100 ng/mL
Immunology and paraprotein studies		
Antinuclear antibodies (ANA)	Negative	<1/160
Anti-myeloperoxidase (MPO-ANCA)	<0.20 IU/mL	Positive >5 IU/mL
Anti-proteinase 3 (PR3-ANCA)	<0.20 IU/mL	Positive >3 IU/mL
C3	98 mg/dL	90.0-180.0 mg/dL
C4	28.4 mg/dL	10.0-40.0 mg/dL
Cryoglobulin screen	Negative	—
Immunoglobulin G (IgG)	347 mg/dL	700-1,600 mg/dL
Serum immunofixation electrophoresis	No monoclonal band	—
Serum free light chain ratio	1.81	0.37-3.1 (adjusted for renal impairment)
HBsAg	Negative	—
Anti-HCV	Negative	—
Anti-HIV 1/2	Negative	—
RPR	Negative	—
Urine studies		
Urine protein (24h)	2,642 mg/24 h	<150 mg/24 h
Urine albumin (24h)	2,210 mg/24 h	<30 mg/24 h
Urine volume	1,500 mL/24 h	—
Urinalysis	Protein +++, leucocyturia, erythrocyturia	—
Urine culture	Negative	—
Urinary Bence Jones protein	Negative	—
Bone marrow findings		
Bone marrow biopsy	CLL/SLL involvement: paratrabecular B-cell aggregates (CD20+, CD5+, CD23+); hypercellular marrow (~60%); suboptimal sample	—

**Table 2:** Chronological summary of the clinical course CLL, chronic lymphocytic leukaemia; eGFR, estimated glomerular filtration rate; iwCLL, International Workshop on Chronic Lymphocytic Leukaemia; MGRS, monoclonal gammopathy of renal significance; PGNMID, proliferative glomerulonephritis with monoclonal immunoglobulin deposits.

Time point	Event	Key clinical, laboratory and pathological findings
-6 months	Baseline	Serum creatinine 1.7 mg/dL. CLL known for 4 years, not meeting iwCLL treatment criteria.
Referral (month 0)	Presentation	Creatinine, 3.3 mg/dL (eGFR, 22 mL/min/1.73 m²); potassium, 6.2 mmol/L; proteinuria, ~2.6 g/24 h; active urinary sediment; lower-limb oedema. Paraprotein studies negative.
+1 month	Diagnostic workup and kidney biopsy	Negative autoimmune, infectious and paraprotein studies. Biopsy: membranoproliferative glomerulonephritis with IgG-κ monoclonal deposits → PGNMID (MGRS).
+1 to +7 months	Treatment	Rituximab + chlorambucil, six 28-day cycles, after multidisciplinary discussion.
+7 months	End of treatment	Creatinine, 1.5 mg/dL (eGFR, ~45 mL/min/1.73 m²); potassium, 4.4 mmol/L; proteinuria, ~0.3 g/24h; oedema resolved.
+12 months	12-month follow-up	Clinically stable and asymptomatic. No haematological relapse or recurrence of proteinuria.

**Figure 1:**
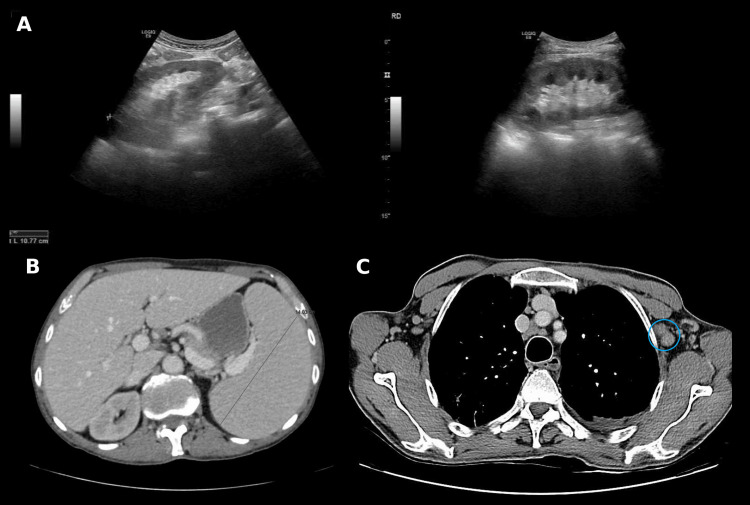
Radiological imaging (A) Renal ultrasound showing the left kidney (left panel) and right kidney (right panel), both normal-sized with diffuse cortical hyperechogenicity. (B) Axial CT of the abdomen demonstrating mild splenomegaly (spleen measuring 14.03 cm). (C) Axial CT of the thorax demonstrating an enlarged left axillary lymph node (14 × 18 mm; blue circle).

A 24-hour urine collection revealed non-nephrotic proteinuria of 2,642 mg/24 h with albuminuria of 2,210 mg/24 h. Urinalysis demonstrated leucocyturia and erythrocyturia; urine culture was negative. Viral serologies for hepatitis B, C, and HIV were negative. Serum protein electrophoresis showed no monoclonal band, urinary Bence Jones protein was undetectable, serum immunofixation electrophoresis revealed no monoclonal immunoglobulin, and serum free light chain ratio was within normal limits (1.81, adjusted for renal impairment), collectively excluding detectable circulating paraprotein. Bone marrow biopsy demonstrated a hypercellular marrow (~60%), with paratrabecular aggregates of small B-lymphocytes expressing CD20, CD5, and CD23, consistent with CLL/small lymphocytic lymphoma (SLL) involvement. The sample was suboptimal (aspiration artefact, core length <1.5 cm); marrow fibrosis could not be evaluated. Kidney ultrasound showed bilaterally normal-sized kidneys with diffuse cortical hyperechogenicity (Figure 1A). CT performed three months prior demonstrated mild splenomegaly (Figure 1B) and a left axillary lymph node of 14 × 18 mm with no other significant lymphadenopathy (Figure 1C).

**Figure 2:**
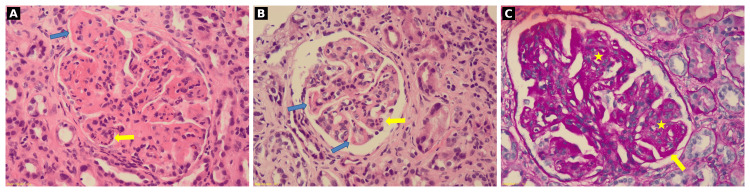
Light microscopy (A) H&E (×10): glomerulus with lobular hypersegmentation, prominent mesangial and endocapillary hypercellularity, and intraluminal neutrophils (yellow arrow); amorphous thickening of the capillary wall with a hyaline appearance (blue arrow) suggests subendothelial deposits. (B) H&E (×10): a second glomerulus with endocapillary hypercellularity and intraluminal neutrophils (yellow arrow) and thickened capillary walls with amorphous hyaline material (blue arrows); a small cellular crescent is partially visible at the glomerular periphery. (C) PAS (×10): glomerulus with lobular hypersegmentation, mesangial expansion, and early nodularity (asterisks); PAS-positive amorphous material within the thickened capillary walls and focal double contours of the glomerular basement membrane (yellow arrow), consistent with subendothelial deposit remodelling. H&E, haematoxylin and eosin; PAS, Periodic acid-Schiff.

Transthoracic echocardiography showed preserved systolic function (left ventricular ejection fraction (LVEF), 62% by Simpson biplane) with no left ventricular hypertrophy or pericardial effusion. Percutaneous kidney biopsy was performed after informed consent. The sample contained up to 13 glomeruli, of which one was globally sclerosed and one showed segmental sclerosis (capsular synechia). Light microscopy revealed a diffuse membranoproliferative pattern with focal nodular glomerulopathy: moderate-to-severe mesangial expansion, at times dense and hyaline, forming PAS-positive, silver-positive, Congo red-negative nodules (a "cotton-wool" appearance), accompanied by moderate-to-severe mesangial hypercellularity (up to 10 mononuclear cells per mesangial area) and hyaline thickening of the capillary walls with double contours and mononuclear cell interposition. Occasional intraluminal neutrophils were present; one glomerulus showed a small cellular crescent and another an endocapillary hyaline thrombus with associated nuclear dust (Figure 2). Tubules showed mild degenerative. Chronic changes were mild (2/10 by the Sethi et al. grading system [6]): global glomerulosclerosis 9%, tubular atrophy 10%, and interstitial fibrosis 10%, with no atherosclerotic lesions. Arterioles were unremarkable; arteries were not represented in the sample. Congo red staining for amyloid was negative.

**Figure 3:**
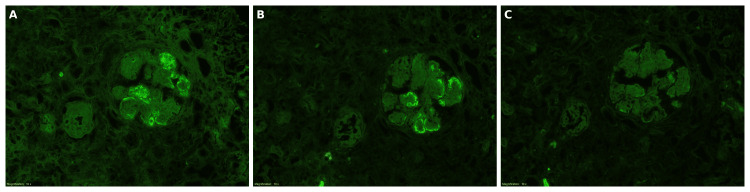
Immunofluorescence (×10) (A) IgG (++++): granular, amorphous, poorly defined deposits in the mesangium and along the glomerular capillary walls, with strong and diffuse staining intensity. (B) Kappa light chain (+++): strong granular mesangial and capillary-wall staining. (C) Lambda light chain (++; scant). The marked predominance of kappa over lambda staining confirms monoclonal kappa light-chain restriction.

Immunofluorescence (frozen sections containing nine glomeruli, none sclerosed) demonstrated granular, amorphous, ill-defined mesangial and capillary-wall deposits of IgG (+++/++++) and kappa light chains (+++/++++), with scant lambda (++) (Figure 3). IgA, IgM, C1q, C3, fibrin, and albumin were all negative. IgG subclass staining was not performed.

**Figure 4:**
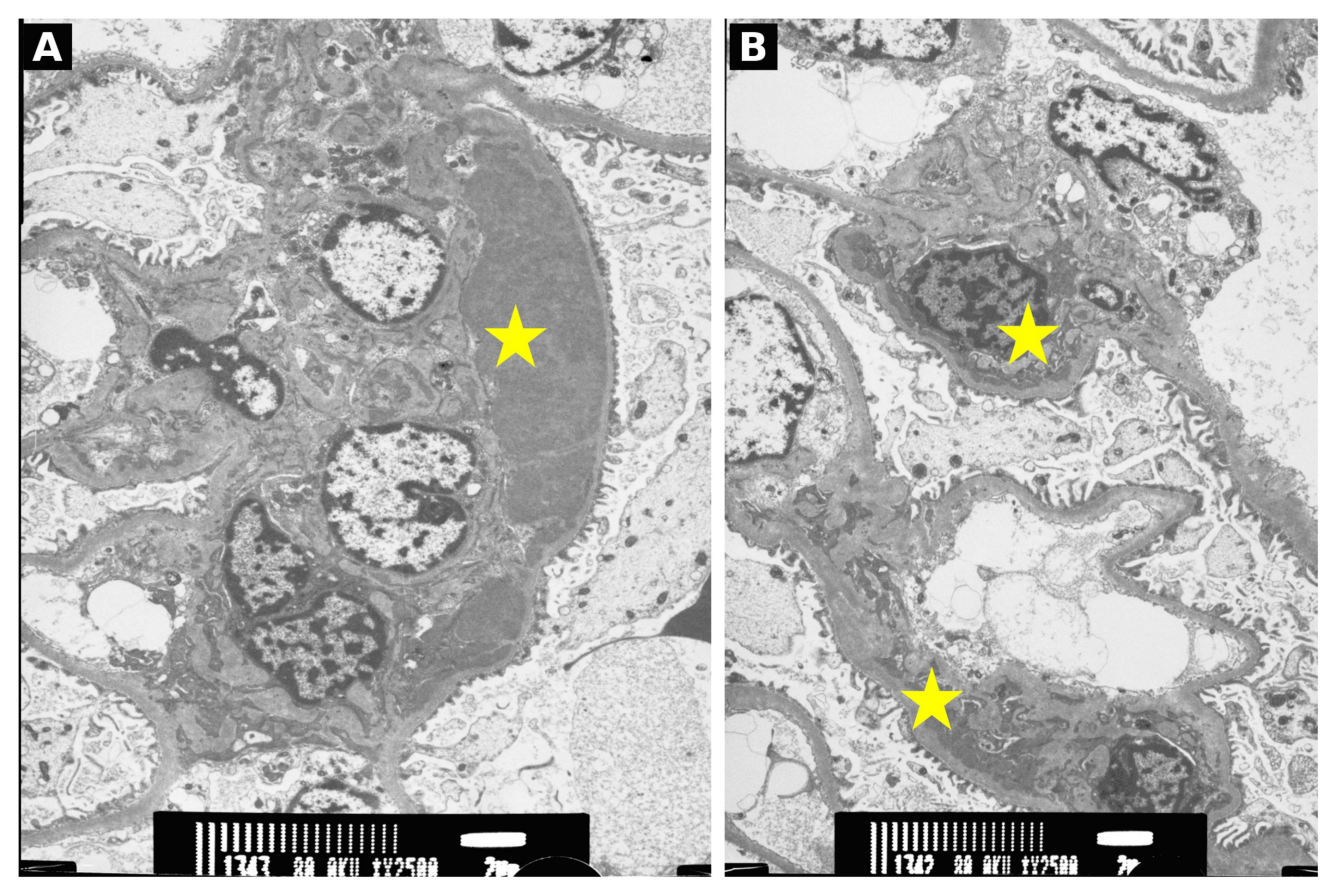
Electron microscopy (×2,500) (A) Amorphous electron-dense deposits in a subendothelial location (asterisk), with no fibrillar or microtubular substructure; the overlying endothelial cell is displaced and the glomerular basement membrane is thickened, consistent with subendothelial monoclonal immunoglobulin deposition. (B) A further example of amorphous electron-dense subendothelial and mesangial deposits (asterisks); the absence of organised substructure excludes fibrillary and immunotactoid glomerulonephritis.

Electron microscopy demonstrated amorphous electron-dense subendothelial and mesangial deposits with no organised fibrillary or microtubular substructure, consistent with PGNMID (Figure 4).

The histological pattern of membranoproliferative glomerulonephritis with kappa-restricted IgG deposits in the context of CLL required systematic exclusion of several overlapping entities. PGNMID was the leading consideration. The kappa-restricted IgG deposits with mesangial and subendothelial distribution, combined with amorphous non-organised deposits on electron microscopy and an underlying monoclonal B-cell clone, were collectively consistent with this diagnosis. IgG subclass typing was not performed; in the general PGNMID population IgG3 predominates, whereas CLL-associated cases more commonly show IgG1, a distinction with potential pathophysiological implications given IgG3's greater propensity for self-aggregation and complement fixation.

Amyloid light-chain (AL) amyloidosis was excluded by the absence of Congo red positivity and non-fibrillar ultrastructural morphology. Fibrillary glomerulonephritis was excluded on the same grounds, with amorphous rather than fibrillar deposit organisation on electron microscopy. C3 glomerulopathy was considered given the membranoproliferative pattern; however, immunofluorescence demonstrated dominant IgG positivity with kappa restriction rather than dominant C3 deposition, and complement levels were not reduced, incompatible with C3 glomerulopathy, in which immunoglobulin staining is characteristically absent or minimal.

Cryoglobulinaemic membranoproliferative glomerulonephritis was considered given the underlying B-cell clone, but serum cryoglobulin testing was negative and there were no systemic features of vasculitis. Infection-related glomerulonephritis was considered in light of endocapillary neutrophilia but excluded by the absence of preceding infection, negative serologies, and the monoclonal immunofluorescence pattern, incompatible with polyclonal immune complex deposits. Integrating the clinical context, the extended negative workup, and the pathological findings, the glomerular disease was attributed to the underlying CLL clone, fulfilling criteria for MGRS.

Following multidisciplinary discussion between Nephrology and Haematology, chronic lymphocytic leukaemia-directed therapy was initiated. The patient commenced rituximab 375 mg/m² intravenously on day 1 of each cycle, combined with chlorambucil 0.5 mg/kg orally on days 1 and 15, over six 28-day cycles, a regimen selected on the basis of the patient’s age, performance status, and comorbidity profile. Hyperkalaemia was addressed prior to starting treatment with dietary potassium restriction and optimisation of diuretic therapy. Supportive measures included antiemetic and antimicrobial prophylaxis, with close monitoring of renal function and electrolytes throughout each cycle. Nephrology follow-up was maintained for the duration of treatment.

Upon completion of the six-month chemoimmunotherapy course, the patient demonstrated marked clinical and biochemical improvement. Serum creatinine fell from 3.3 mg/dL at presentation to 1.5 mg/dL, with a corresponding improvement in estimated glomerular filtration rate from 22 to approximately 45 mL/min/1.73 m². Hyperkalaemia resolved, with serum potassium normalising to 4.4 mmol/L. Proteinuria fell from approximately 2.6 g/24 h at presentation to approximately 0.3 g/24 h. Lower limb oedema resolved completely, and the patient reported a return to his usual level of daily activity. Haematological response paralleled the renal improvement. By the end of treatment, the white cell count had fallen from 62.1 to 11.1 × 10⁹/L, with the absolute lymphocyte count decreasing from approximately 54 to 3.6 × 10⁹/L. Haemoglobin rose to 11.3 g/dL (mean corpuscular volume (MCV), 97 fL) and the platelet count normalised to 216 × 10⁹/L. Cross-sectional imaging demonstrated regression of the previously documented splenomegaly and left axillary lymphadenopathy.

At 12-month follow-up, the patient remains clinically stable and asymptomatic. He is under regular review in both Nephrology and Haematology outpatient clinics, with serial assessment of renal function, urinary protein excretion, serum electrolytes, full blood count, and haematological response markers. Follow-up is conducted in accordance with European Society for Medical Oncology and Kidney Disease Improving Global Outcomes guidance for treated chronic lymphocytic leukaemia and chronic kidney disease, respectively [2,7]. No relapse of haematological disease or recurrence of proteinuria has been documented to date. Given the degree of chronic tubulointerstitial injury identified on biopsy, long-term decline in kidney function remains a possibility and continued nephrology surveillance is planned. The patient has been informed of this risk and is engaged in shared decision-making regarding ongoing care.

## Discussion

This case illustrates an uncommon but clinically significant renal complication of CLL: immune-mediated glomerulonephritis resulting in progressive kidney failure. Renal manifestations may precede, coincide with, or follow the haematological diagnosis, and are increasingly recognised within onco-nephrology [1,2]. In a landmark Mayo Clinic series of 49 patients with CLL who underwent kidney biopsy, membranoproliferative glomerulonephritis was the most frequently identified pattern, followed by PGNMID and cryoglobulinaemic glomerulonephritis [3]. Consistent findings across smaller series include circulating monoclonal protein detectable in only ~30% of cases, frequent need for biopsy to establish diagnosis, and renal improvement with clone-directed therapy in the majority of treated patients [4].

The findings in this patient are consistent with PGNMID within the MGRS spectrum. The proposed mechanism involves secretion of a nephrotoxic monoclonal immunoglobulin by the CLL clone, depositing in the glomerular mesangium and along capillary walls, activating complement and driving endocapillary inflammation [4,8]. IgG kappa restriction, as in this case, is the most commonly reported immunofluorescence pattern in PGNMID [9]. Paraprotein was undetectable by serum protein electrophoresis, immunofixation, and free light chain assay (ratio 1.81, within normal limits adjusted for renal impairment), consistent with published data and further underscoring the indispensable role of kidney biopsy with full immunofluorescence and electron microscopy characterisation, as recommended by the International Kidney and Monoclonal Gammopathy Research Group [5].

Beyond establishing aetiology, the biopsy served a second critical function: by documenting symptomatic organ involvement, it fulfilled an iwCLL criterion for active disease, providing the haematological indication to initiate systemic therapy in a patient previously managed expectantly [2]. This is the clinical scenario for which CLL with renal significance has been proposed: an untreated clone causing measurable end-organ damage that mandates intervention [1]. Rituximab combined with chlorambucil was selected in accordance with MGRS guidance for CLL-type clones and European Society for Medical Oncology (ESMO) guidelines supporting anti-CD20-based regimens as first-line treatment in older or less fit patients [4,6,10]. Without treatment, PGNMID follows a natural history of progressive kidney function decline; early clone-targeted intervention is therefore critical to preserve residual nephron mass [9].

The outcome, creatinine falling from 3.3 to 1.5 mg/dL and proteinuria reducing to 0.3 g/24 hours at 12-month follow-up, supports a causal relationship between the CLL clone and the glomerular injury and is consistent with published series. Three limitations merit acknowledgement. First, the bone marrow biopsy was suboptimal (core <1.5 cm), potentially underestimating CLL marrow involvement. Second, IgG subclass typing was not performed; the distinction between IgG3 (predominant in the general PGNMID population) and IgG1 (more common in CLL-associated cases) would have added pathophysiological granularity. Third, although the renal response to clone-directed therapy strongly supports a pathogenic link, the monoclonal nature of the glomerular deposits was established immunohistologically (IgG-kappa restriction) rather than by direct molecular demonstration that the deposited immunoglobulin originated from the CLL clone. Should re-treatment be required, salvage therapy carries specific nephrotoxic considerations: ibrutinib is associated with hypertension, atrial fibrillation, and prothrombotic effects; venetoclax carries a risk of tumour lysis syndrome amplified by impaired renal clearance, requiring careful dose escalation and close nephrological monitoring.

## Conclusions

Renal involvement in CLL may manifest as progressive kidney injury and proteinuria in the absence of detectable circulating paraprotein. Kidney biopsy with immunofluorescence and electron microscopy is therefore indispensable, both to establish the diagnosis of PGNMID and, by documenting symptomatic organ involvement, to trigger clone-directed therapy through fulfilment of iwCLL criteria. The marked renal response to rituximab and chlorambucil strongly supports a probable causal relationship between the CLL clone and the glomerular injury, although direct molecular proof linking the renal deposits to the clone was not available. A low threshold for kidney biopsy should be maintained in CLL patients with an unexplained decline in eGFR, proteinuria, haematuria, an active urinary sediment, or oedema, particularly when conventional aetiological workup is unrevealing.
